# Molecular Mechanisms Regulating Hepcidin Revealed by Hepcidin Disorders

**DOI:** 10.1100/tsw.2011.130

**Published:** 2011-07-07

**Authors:** Clara Camaschella, Laura Silvestri

**Affiliations:** Vita-Salute University and Division of Genetics and Cell Biology, San Raffaele Scientific Institute, Milan, Italy

**Keywords:** iron, iron metabolism, hepcidin, erythropoiesis, anemia

## Abstract

Iron is essential for human life, but toxic if present in excess. To avoid iron overload and maintain iron homeostasis, all cells are able to regulate their iron content through the post-transcriptional control of iron genes operated by the cytosolic iron regulatory proteins that interact with iron responsive elements on iron gene mRNA. At the systemic level, iron homeostasis is regulated by the liver peptide hepcidin. Disruption of these regulatory loops leads to genetic diseases characterized by iron deficiency (iron-refractory iron-deficiency anemia) or iron overload (hemochromatosis). Alterations of the same systems are also found in acquired disorders, such as iron-loading anemias characterized by ineffective erythropoiesis and anemia of chronic diseases (ACD) associated with common inflammatory conditions. In ACD, iron is present in the body, but maldistributed, being deficient for erythropoiesis, but sequestered in macrophages. Studies of the hepcidin regulation by iron and inflammatory cytokines are revealing new pathways that might become targets of new therapeutic intervention in iron disorders.

### INTRODUCTION

Iron is an essential element for life since it modulates fundamental processes, such as hemoglobin synthesis, oxygen transport, and cell respiration and proliferation. However, due to its propensity to release electrons and produce reactive oxygen species, excess iron is toxic. For this reason, several regulatory mechanisms have been developed in mammals to avoid iron overload and to regulate iron uptake, utilization, release, and storage, according to cell and organism needs[1]. At the cellular level, this regulation occurs through the well-known IRE-IRP system (iron responsive elements – iron regulatory proteins); at the systemic level, through the modulation of expression of the iron hormone hepcidin[1]. Disruption of these regulatory loops leads to genetic (and acquired) disorders, characterized by iron deficiency, iron overload, or maldistribution.

### The Iron Regulator Hepcidin

Hepcidin, the central iron regulator, is a small peptide produced by the hepatocytes in response to increased body iron and inflammation. Hepcidin binds to the iron exporter ferroportin on duodenal enterocytes and macrophages, triggering its internalization and lysosomal degradation[2]. In this way, circulating hepcidin controls both intestinal iron absorption and the release of iron from macrophages into plasma with a negative feedback mechanism[3]. Although other players have been identified recently that modulate intestinal iron absorption, such as HIF2-alpha[4] or ferritin H[5], hepcidin remains the central regulator of iron homeostasis, as demonstrated by the genetic disorders that follow its inactivation or overexpression. Iron-dependent hepcidin activation in the hepatocytes requires signaling through the bone morphogenic proteins (BMPs) – sons of mothers against decapentaplegic (SMAD) pathway (Fig. 1). BMP6 has been shown to be the iron-sensitive BMP[6,7,8] that binds to its receptors (BMPR type I and II) with the essential assistance of the coreceptor hemojuvelin (HJV)[9]. This binding triggers a SMAD-dependent signaling cascade in hepatocytes. Recent data suggest that BMP6 in normal iron status is mainly expressed by nonparenchymal cells (Kupffer, stellate, sinusoidal endothelial cells) of the liver[10].

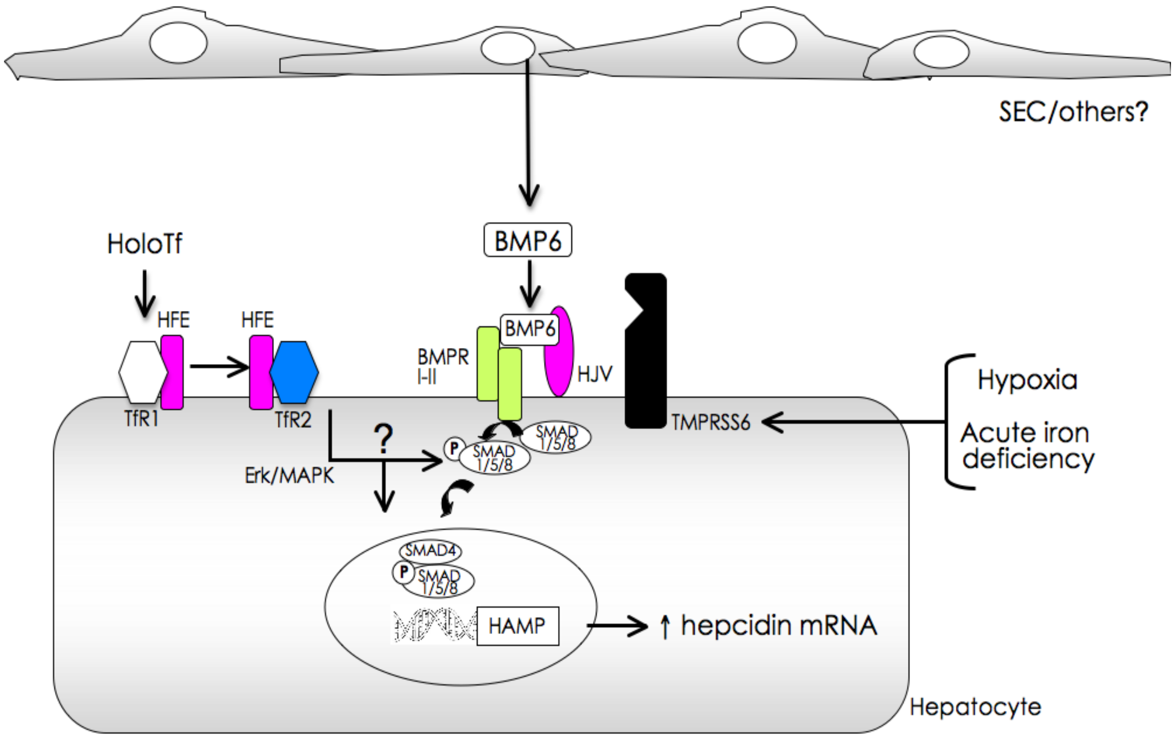

**FIGURE 1.** Modulation of hepcidin transcription by iron. Diferric (Holo) transferrin binds to its receptor TfR1 and leaves HFE free to interact with TfR2, which is stabilized on the cell surface. Interaction between HFE and TfR2 modulates hepcidin transcription through a mechanism not yet fully clarified. BMP6 is produced mainly by SEC (sinusoid endothelial cells) and other nonparenchymal cells[10], and is transcriptionally activated by intracellular iron increase. The hepatocyte-specific BMP coreceptor HJV binds to BMP receptors type I and II (BMPRI-II) in the presence of the ligand BMP6. Formation of the multiprotein complex on the cell surface of hepatocytes activates the phosphorylation of SMAD1/5/8 and their interaction with SMAD4. The SMAD complex translocates to the nucleus for hepcidin activation. Conditions of hypoxia/acute iron deprivation increase the activity of the transmembrane serine protease TMPRSS6, which impairs hepcidin mRNA by cleaving membrane HJV.

Hepcidin expression is enhanced by inflammatory cytokines, especially IL-6, which binds to its receptor, activating JAK2 signaling and STAT3 phosphorylation[11]. The inflammatory pathway requires the integrity of the BMP-SMAD pathway to fully activate hepcidin[12].

The BMP-SMAD pathway and hepcidin transcription are both down-regulated in hypoxia, iron deficiency, and erythropoietic expansion, but the molecular mechanisms of this down-regulation are unclear[1]. While BMPs play a crucial role in the up-regulation of hepcidin expression, the serine protease matriptase-2 encoded by TMPRSS6[13,14,15] is essential in hepcidin inhibition (see below).

The hepcidin identification has dramatically changed our understanding of the systemic regulation of iron metabolism and the pathophysiology of inherited iron disorders, now explained based on hepcidin dysregulation. In addition, it has allowed the recognition of new disorders, such as iron-refractory iron-deficiency anemia (IRIDA)[13,14,15], due to the lack of the hepcidin inhibitory TMPRSS6. At the same time, elucidating genetic iron disorders has contributed to further understanding of hepcidin regulation and has highlighted some unsolved questions. Finally, the identification of hepcidin and its pathway have provided new molecular targets for the development of novel therapeutic approaches for iron disorders[16].

### GENETIC DISORDERS OF THE HEPCIDIN PATHWAY

#### Disorders Caused by Hepcidin Deficiency

Hepcidin deficiency leads to excessive ferroportin function and systemic iron overload, with iron accumulation and toxicity in parenchymal cells of the liver and other organs. Genetic iron overload (hereditary hemochromatosis) was first described in the 19$^{th}$ century as “bronze diabetes” because of the recognizable clinical complications due to excess tissue iron deposition, i.e., diabetes and dark skin. At present, using genetic tests, the hemochromatosis genotype may be diagnosed as early as at the stage of genetic susceptibility to develop iron overload, before the development of any clinical complications[17]. Hemochromatosis is genetically heterogeneous, caused by at least five different genes (Table 1). HFE hemochromatosis is the most frequent form; most patients are homozygous for the HFE C282Y mutation. HFE is an atypical major histocompatibility complex class I–like protein ubiquitously expressed, which heterodimerizes with beta2-microglobulin for cell surface localization[18]. HFE associates with the transferrin receptor (TfR1) and participates in its endosomal cycle[19], in competition with diferric-transferrin (Fe2-Tf) (Fig. 1). In the present model, when Fe2-Tf increases, HFE dissociates from TfR1 and forms a complex with TfR2[20,21]. The latter complex is proposed to activate hepcidin transcription in response to the increased circulating Fe2-Tf, but the molecular mechanisms remain elusive (reviewed in Hentze et al.[1]). This effect does not occur in hemochromatosis, since HFE C282Y is not expressed on the cell surface since it cannot bind beta2-mycroglobulin. Recently it has been shown in mice that Hfe is important for a full activation of the SMAD pathway since its deficiency is associated with a reduced SMAD1/5/8 phosphorylation[22,23] in the presence of the congruent increase of BMP6 expression.

Juvenile hemochromatosis is a recessive hemochromatosis due to HJV mutations. HJV is a GPI-linked membrane protein, which is a BMP coreceptor essential for iron-mediated hepcidin activation[9]. Some HJV mutations decrease the coreceptor expression on the cell surface[24]; others abolish the interaction with BMPs ([25] and our unpublished results) and are unable to activate hepcidin *in vitro*[26]. Hepcidin mutations are extremely rare. They lead to the virtual absence of the protein or affect the invariable cysteines, and thus the peptide hairpin conformation. All defects cause an early-onset, severe phenotype identical to that of the relatively more common *HJV* mutations[27], also characterized by extremely low hepcidin levels[28]. The toxicity of iron in juvenile hemochromatosis is likely dependent on the BMP pathway down-regulation, which favors a maximal rate of iron accumulation.

**TABLE 1**
**Classification of Hepcidin Disorders**

| | Gene | Inheritance | Mechanism | Phenotype |
|---|---|---|---|---|
| **Genetic hepcidin deficiency** | | | | |
| Hemochromatosis type 1 | HFE | AR | Defective hepcidin activation | Iron overload |
| Hemochromatosis type 2A | HJV | AR | Lack of BMP coreceptor | Iron overload |
| Hemochromatosis type 2B | HAMP | AR | Lack of hepcidin | Iron overload |
| Hemochromatosis type 3 | TfR2 | AR | Defective hepcidin activation | Iron overload |
| **Genetic hepcidin resistance** | | | | |
| Hemochromatosis type 4B | FPN | AD | FPN resistant to hepcidin function | Iron overload |
| **Genetic defect of the hepcidin receptor** | | | | |
| Hemochromatosis type 4A (ferroportin disease) | FPN | AD | Loss of FPN function | Defective iron recycling |
| **Genetic hepcidin overexpression** | | | | |
| IRIDA | TMPRSS6 | AR | Defective hepcidin inhibition | Iron-refractory anemia |
| **Acquired hepcidin deficiency** | | | | |
| Iron-loading anemias | | | Excessive hepcidin inhibition | Anemia, iron overload |
| **Acquired hepcidin overexpression** | | | | |
| Hepcidin-producing adenoma | | | Inappropriate hepcidin expression | Microcytic anemia |
| Inflammation (acute, chronic) | | | IL-6–induced hepcidin expression | Anemia of inflammation, ACD |

*TfR2*-hemochromatosis is a further type of recessive disease, whose expression (early onset, but moderate severity) differs from both *HFE*-related and juvenile hemochromatosis, suggesting that TfR2 exerts more complex functions than that of a simple HFE partner. In agreement, *Hfe*-/- mice have less severe iron overload than *Tfr2*-/- mice and the double knockout has the most severe phenotype[29]. The recent identification of TfR2 as a partner of the erythropoietin receptor in the bone marrow[30] makes the interpretation of its function more complex and suggests that TfR2 may represent a potential link between erythropoiesis, erythropoietin, and hepcidin.

Ferroportin is the functional receptor of hepcidin and is essential for life[31]. Diseases of ferroportin have dominant inheritance. Heterozygous mutations cause hemochromatosis type IV also called “ferroportin disease”. The typical disorder is different from hemochromatosis: loss-of-function mutations, which reduce the surface expression of ferroportin, cause macrophage iron retention and restrict iron available for erythropoiesis[32]. However, few gain-of-function mutations render ferroportin resistant to the hepcidin effect[33], either because of loss of hepcidin binding or of ferroportin internalization. In these cases, mutant ferroportin remains on the macrophage surface and contributes to increase the available iron, causing the unique form of hepcidin-resistant hemochromatosis with high hepcidin levels[34].

Animal models are available for all hemochromatosis types (for review, see Supplemental Table 1 in Hentze et al.[1]). It must be emphasized that murine models do not recapitulate all disease complications, but are useful to study mechanisms of iron loading that are conserved between mice and humans. Major advances have been obtained by these models; for example, only selective inactivation of *Hfe* in hepatocytes causes iron overload[35], whereas inactivation of *Hfe* in the duodenum[35] or macrophages[35] does not change the iron status. Inactivation of *Tfr2* causes iron overload that is even more severe in selective hepatocyte inactivation[36,37]. Compared to these models, inactivation of hepcidin[38] or HJV[39,40] causes earlier and more severe iron overload of the liver, heart, and pancreas, reminiscent of iron accumulation in juvenile hemochromatosis[1]. A spontaneous mouse strain *(flatiron* mice) has a mutation in ferroportin and provided the first genetic model of ferroportin disease, also showing that it results from dominant-negative effects rather than haploinsufficiency[41].

Some murine models of iron overload due to extremely low hepcidin levels have been described that do not have a human correspondent, such as beta2-microglobulin knockout[42], *Bmp6*-/-[8], or liver-conditional *Smad4* knockout mice[12]. The beta2-microglobulin knockout mouse[42] was the first murine model of iron overload reported. It occurs because beta2-microglobulin is a partner of the HFE protein on the cell surface. *Bmp6*-/- mice were more recently identified as a model of severe hemochromatosis since Bmp6 is the major activator of hepcidin expression[8]. Liver-conditional *Smad4* knockout mice[12] have severe iron overload and are unable to increase hepcidin, not only in response to iron, but also to inflammatory cytokines[12], demonstrating that Smad4 represents a critical checkpoint of hepcidin activation and a site of cross-talk between inflammatory and iron pathways.

Bmp6 is up-regulated in response to iron overload in *Hfe*-/- mice, but Smad signaling and hepcidin levels are down-regulated[22,23], indicating that Hfe likely plays a role in the Bmp-Smad signaling pathway. Transgenically expressed *Hfe* does not correct the iron overload of *Hjv* knockout mice, demonstrating that Hfe is not downstream Hjv. Double knockout for *Hfe* and *Tmprss6* (the liver-specific serine protease that down-regulates hepcidin through the cleavage of membrane Hjv[43]) have iron deficiency with high hepcidin, while heterozygous loss of *Tmprss6,* increasing the Bmp/Smad signaling, partially corrects the iron overload of *Hfe*-/- mice[44]. These data exclude that Hfe is downstream Tmprss6.

In conclusion, hemochromatosis results from the interruption of the regulatory axis that activates hepcidin in response to increased body iron. A unifying model relies on hepcidin dysregulation, either decreased hepcidin in recessive diseases due to mutations of *HFE*, *TfR2*, *HJV*, *hepcidin*, or due to hepcidin resistance in selected ferroportin mutations.

### Disorders Caused by Hepcidin Excess

Hepcidin overexpression was first shown to cause iron deficiency anemia in hepcidin transgenic mice[45]. More recently, it was demonstrated in two engineered animal models both unable to inhibit hepcidin expression: the “Mask” mouse[13] and the *Tmprss6* knockout mouse[14]. Both models lack Tmprss6 function. *Tmprss6* in the Mask mouse encodes a truncated inactive protein devoid of the serine protease domain because of a splicing defect[13]. The Mask mice, characterized by gradual loss of body, but not facial, hair, are smaller than their littermates and have microcytic anemia, low plasma iron, and depleted iron stores. Moreover, homozygous females are infertile[13].

Molecular defects inactivating TMPRSS6 cause the inherited disorder called iron-refractory iron-deficiency anemia (IRIDA) in humans (Table 1). IRIDA is a rare, recessive condition described in few families worldwide[46]. Usually recognized in children, IRIDA is characterized by a moderate degree of anemia, extreme microcytosis and hypochromia, low transferrin saturation, and normal/high hepcidin levels[46]. In cases followed for many years, the severity seems to decrease over time, paralleling the reduced iron requirements of adult life[46,47]. Recent developments in hepcidin dosage[48,49,50] and international standardization efforts[51] will facilitate the disease characterization in the future because, except when inflammatory conditions coexist, iron-deficient subjects have usually low/undetectable hepcidin levels in serum and/or urines.

## HEPCIDIN IN ACQUIRED DISORDERS

### Hepcidin Deficiency in Acquired Disorders

Hepcidin deficiency was first reported in beta-thalassemia patients and then shown to be a feature of the so-called “iron-loading anemias”. These anemias are characterized by high degrees of ineffective erythropoiesis and high iron stores. However, despite iron overload, hepcidin is not increased. They are considered a model to study the “erythroid regulator”. This concept was defined by Clement Finch more than 30 years ago to indicate the strong signal that leads to increased iron absorption according to the erythropoiesis needs, irrespective of the iron stores[52]. Reasonably in this condition, the BMP-SMAD major activating pathway should be down-regulated. A proposed mechanism relies on the effect of cytokines of the TGF-beta family released by the erythroblasts. Candidate mediators are growth differentiation factor 15 (GDF15) released by mature[53] and twisted gastrulation protein homolog 1 (TWGS1)[54] released by immature (murine) erythroblasts. However, a convincing role of these cytokines in this process is not demonstrated. Extremely high levels of GDF15 in beta-thalassemia could be accounted for by the huge ineffective erythropoiesis, but it may be also released by other hypoxic cells. The levels of GDF15 that are able to suppress hepcidin transcription in hepatocyte cultures (and less efficiently in hepatoma cell lines)[53] are not observed in other conditions of ineffective erythropoiesis, such as congenital dyserythropoietic anemia type I[55]. Searching for another player, the same authors proposed(TWGS1, a potential ligand of BMPs released by immature erythroblasts in the thalassemic mouse, but its role in humans remains unproven[54]. Other candidates for at least partial hepcidin suppression are the hypoxia mediator HIF-1alpha[56], or the increased erythropoietin expression with direct[57] or more likely indirect[58] effect. Another important player is the serine protease TMPRSS6, which down-regulates hepcidin by cleaving membrane HJV[43]. TMPRSS6 transcription is increased *in vitro* in hypoxia[59] and might inhibit hepcidin, decreasing the BMP coreceptor HJV on the cell surface[43]. Also, the BMP ligand, soluble HJV, which has been shown to be increased by furin cleavage in hypoxia/iron deficiency in cell culture[60], might play a role as a BMP antagonist.

Other conditions in which hepcidin is reduced are severe liver disease and alcoholic liver disease, characterized by iron overload, due to insufficient hepcidin production. Moderate hepcidin decrease in chronic viral C hepatitis has been reported[61] due to viral-mediated suppression of hepcidin production[62].

### Hepcidin Excess in Acquired Disorders

Rare cases of hepcidin-producing, benign, hepatic adenomas have been reported in patients treated for inherited glycogen storage disease type 1a caused by glucose-6-phosphatase-deficiency[63]. All patients had microcytic anemia, with features of iron deficiency, but normal ferritin levels. Anemia reverted after surgery that removed the hepcidin-expressing adenoma. This rare disorder and IRIDA underline that persistently high hepcidin causes microcytosis.

More commonly, hepcidin overexpression occurs in anemia of chronic diseases (ACD) or anemia of inflammation. This is a common form of anemia observed in infections/inflammatory disorders associated with overproduction of inflammatory cytokines, especially IL-1b, TNF-alpha, IL-6, and gamma-IFN[64]. Anemia is multifactorial in inflammatory conditions: blunted erythropoietic response to erythropoietin (which is decreased during inflammation) and reduced erythrocyte survival coexist with iron abnormalities, such as macrophage iron sequestration and iron-restricted erythropoiesis. Transferrin saturation is normal/low and serum ferritin normal/high. The iron abnormalities are explained by inappropriately high hepcidin expression enhanced by IL-6–dependent activation of the STAT3 pathway. Hyperproduction of hepcidin in inflammation also occurs in macrophages in response to IL-6 and toll like receptor 4 stimulation[65]. This might amplify iron retention through the autocrine effect of hepcidin on macrophage ferroportin. In addition, according to recent results, hepcidin production could be beneficial to stimulate an anti-inflammatory response[66]. Because macrophages play a major role in recycling iron for erythropoiesis, insufficient iron release leads to anemia. However, red cells usually are not microcytic, unless the process is longstanding or true iron deficiency coexists[64]. ACD differs from both iron deficiency and overload, and can be classified as a defect of iron recycling (Table 1) or a condition of iron maldistribution.

Anemia of cancer has been referred to inflammatory-mediated hyperproduction of hepcidin, in particular in multiple myeloma[67], in Hodgkin's lymphoma[68], and Waldenstrom macroglobulinemia[69]. Hepcidin has been demonstrated to be expressed in breast cancer tissue: a recent paper based on expression arrays in breast cancer reports that the hepcidin/ferroportin ratio is a potential novel and independent prognostic marker in large series of breast cancer patients[69], implying the hepcidin production by cancer tissue. However, the specific cell type that produces hepcidin remains to be demonstrated and whether hepcidin is produced by cancer-recruited inflammatory macrophages remains to be excluded[70]. Iron certainly has an essential role in cancer, but whether tumor cells other than hematopoietic cells[70] may produce hepcidin with an autocrine effect to favor cancer growth remains to be demonstrated.

Iron is essential for all cells of the body and obviously its role is not limited to erythropoiesis or liver disease. We are at the beginning of understanding the role of iron and its regulatory pathways in other broad fields of medicine, including atherosclerosis, susceptibility to infections, renal insufficiency, and neurodegenerative disorders. Future years will see a further expansion of the role of iron in biology and medicine.

### ACKNOWLEDGMENTS

This work was partially supported by grants from eRARE 2009 (HMA-Iron) and Cariplo, Milan, Italy 2009-2483.
